# The hotspots and publication trends in postoperative delirium: A bibliometric analysis from 2000 to 2020

**DOI:** 10.3389/fnagi.2022.982154

**Published:** 2022-09-26

**Authors:** Xiaowan Lin, Ying Cao, Xiao Liu, Kang Yu, Huihui Miao, Tianzuo Li

**Affiliations:** Department of Anesthesiology, Beijing Shijitan Hospital, Capital Medical University, Beijing, China

**Keywords:** POD, elderly, bibliometric, hotspots, publication trends

## Abstract

**Background:**

Postoperative delirium (POD) is a common aging-associated postoperative complication that has received increasing attention in the context of the aging global population and the number of articles published on POD is gradually increasing. This study aimed to quantify the basic information of scholarly publications on POD and identify the most impactful literature, trends, and hotspots in POD research.

**Materials and methods:**

We searched articles on POD through the Science Citation Index Expanded databases published from 2000 to 2020. Bibliographic information, including year, country, authorship, type, journal, funding, affiliations, subject areas, and hotspots, was collected for further analysis.

**Results:**

A total of 2,114 articles on POD from 2000 to 2020 were identified. The highest number of studies (*n* = 748) were published in the United States, comprising the most total citations (13,928), followed by China (*n* = 278), and Germany (*n* = 209). Inouye, Sharon K. was the most productive author, with 66 publications on POD. The *Journal of the American Geriatrics Society* published the highest number of articles (*n* = 80), with the most total citations (4,561) and average (57.01), followed by *Anesthesia and Analgesia* (*n* = 52), and the *British Journal of Anaesthesia* (*n* = 43). Harvard University was the most productive institute, with the highest H-index (*n* = 46) and highest degree centrality (*n* = 191). The top hotspots in the field of POD during this period were “elderly,” “cardiac surgery,” “cognitive impairment,” “hip fracture,” and “intensive care unit.”

**Conclusion:**

This study provides an overview of developments in the field of POD over the past 20 years using bibliometric analysis. Overall, research on POD has flourished worldwide. The United States (US) has a relatively high academic impact owing to its productive expertise and institutions in this field. Despite much research illustrating the diagnosis and management of POD in clinical practice, more basic research is needed.

## Introduction

Postoperative delirium (POD) is a common complication that is characterized by acute and fluctuating changes in mental status, attention, and level of consciousness after surgeries and anesthesia (Aldecoa et al., 2017). According to the recommendations for the nomenclature of cognitive change associated with anesthesia and surgery in 2018, by the nomenclature consensus working group, POD was included in perioperative neurocognitive disorder (PND); however, should be recognized as a specific category that is consistent with the diagnostic criteria (Diagnostic and Statistical Manual for Mental Disorders, fifth edition, DSM-5) (Evered et al., 2018). Before the nomenclature, POD was always studied as an independent concept since the acute clinical symptoms were different from those of postoperative cognitive dysfunction (POCD). The incidence of POD ranges from 5 to 50% and can occur at any age; however, mostly in the elderly (American Geriatrics Society Expert Panel on Postoperative Delirium in Older Adults, 2015). It has been demonstrated that POD was associated with adverse outcomes, such as increased mortality, prolonged hospitalization, increased incidence of POCD, and higher medical cost (Bai et al., 2020; Hughes et al., 2020). Numerous studies have aimed to identify the risk factors for POD, including advanced age, multiple comorbidities, preoperative cognitive impairment, poor vision or hearing, and presence of infection (American Geriatrics Society Expert Panel on Postoperative Delirium in Older Adults, 2015; Raats et al., 2015; Yang et al., 2017). However, the pathogenesis of POD has not been fully elucidated, and the research for more effective treatments is ongoing. Meanwhile, with the trend of global aging, POD, being an aging-associated disease, has attracted increasing attention from researchers. Therefore, we analyzed publications and hotspots in the field of POD in this study.

Bibliometric analysis is a method of analyzing books, articles, and other documents using mathematical and statistical techniques (Roldan-Valadez et al., 2019), allowing researchers to gain a general understanding of the frontier area and hotspots in a certain field. Bibliometric analysis also provides a reference for accurately reading the literature and for selecting research directions. It has been widely applied in many fields (Hong et al., 2019; Mi et al., 2021), including hotspots in POCD and the hundred most cited articles in PND (Chen et al., 2020; Mi et al., 2021); however, few bibliometric studies have been performed on POD. This study aimed to quantify the basic information of scholarly publications and identify the most impactful literature, trends, and hotspots, thereby providing a comprehensive overview of the current status of POD research.

## Materials and methods

### Search strategy

**FIGURE 1:**
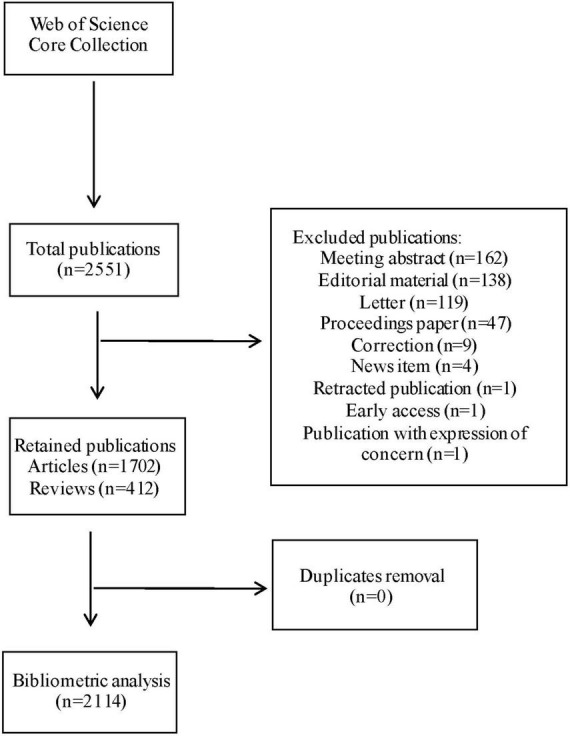
Flowchart for the publication selection included in this study.

An online literature search was performed on May 19, 2021, using the Science Citation Index Expanded databases. Articles and reviews published between 2000 and 2020 were included in this analysis. The requirement for written informed consent was waived by the Institutional Review Board. This manuscript reports results of an observational bibliometric study, and therefore follows the applicable Enhancing the QUAlity and Transparency Of health Research EQUATOR guidelines (PRISMA and STROBE). The terms used in the search were SU = (POD) and Language = English. Figure 1 showed the detail of the search flow. A total of 2,551 records were found, excluded publications were meeting abstract (*n* = 162), editorial material (*n* = 138), letter (*n* = 119), proceedings paper (*n* = 47), correction (*n* = 9), news item (*n* = 4), retracted publication (*n* = 1), early access (*n* = 1), and publication with expression of concern (*n* = 1). Then, duplicates removal (*n* = 0) was performed. Finally, The retained publications (*n* = 2114) with 1,702 articles and 412 reviews were employed for bibliometric analysis. The following information was collected: year, country, authorship, type, journal, funding, affiliations, subject areas, and hotspots. Figure 1 shows the search flow for this bibliometric-analysis.

### Statistical analysis

The CiteSpace software was used for bibliometric analysis (co-citation, co−occurrence, hotspots, and cluster analysis). Statistical analyses were performed using SPSS version 23 (IBM SPSS, Inc., Chicago, IL, USA). Data were expressed as numbers or percentages. Correlation analysis was performed using a two-tailed Pearson’s correlation. Statistical significance was set at *P* < 0.05.

## Results

### Analysis of years and countries

A total of 2,114 articles were employed in our search strategy. The United States (US) was top-ranked with the highest number of publications on POD (*n* = 748), followed by China (*n* = 278), Germany (*n* = 209), and Canada (*n* = 148). The US also had the highest total citations (13,928), followed by Germany (3,660), England (3,616), Canada (3,591), and the Netherlands (3,546). Inconsistent with the number of articles, Denmark had the highest mean number of citations per paper (32.38), followed by Belgium (31.96), Netherlands (29.07), Sweden (29.00), and Ireland (27.42; Table 1).

**TABLE 1:** The top 20 countries with the highest number of publications on postoperative delirium (POD).

Rank	Country	Number of papers	Total citations	Mean citations/ Paper
1	USA	748	13,928	18.62
2	China	278	2,563	9.22
3	Germany	209	3,660	17.51
4	Canada	148	3,591	24.26
5	Japan	141	1,635	11.60
6	England	137	3,616	26.39
7	Netherlands	122	3,546	29.07
8	Australia	91	1,718	18.88
9	South Korea	78	739	9.47
10	Italy	79	1,523	19.28
11	Switzerland	51	1,372	26.90
12	Sweden	43	1,247	29.00
13	Denmark	42	1,360	32.38
14	Poland	36	529	14.69
15	France	34	497	14.62
16	Spain	33	664	20.12
17	Belgium	26	831	31.96
18	Ireland	26	713	27.42
19	Turkey	26	357	13.73
20	Norway	24	494	20.58

**FIGURE 2:**
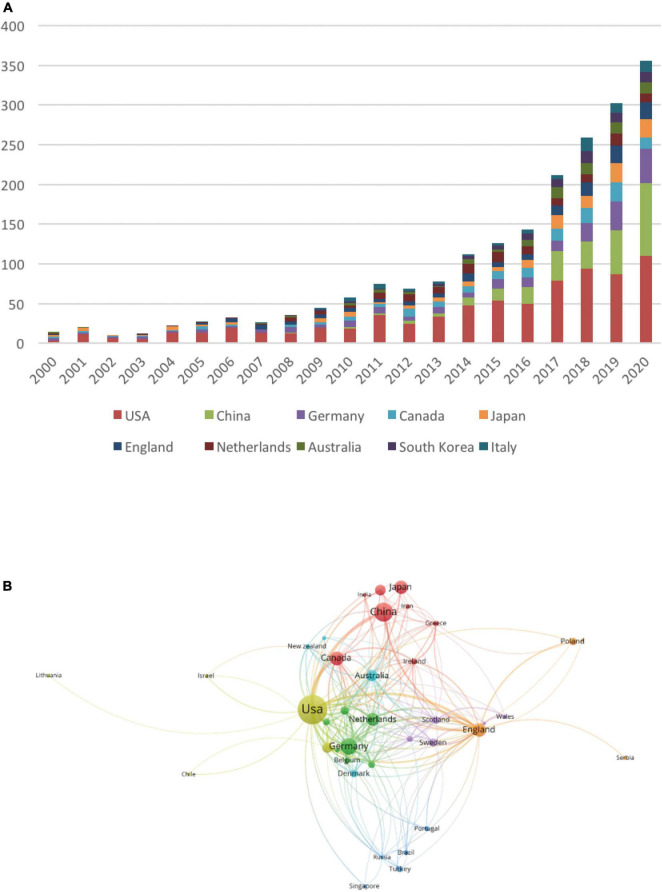
The effect of changes in years and countries on the number of postoperative delirium (POD) articles. **(A)** The number of POD articles from the top 10 countries per year. **(B)** The network visualization map indicating collaborations between countries.

The total number of articles from the top 10 countries increased annually from 2000 to 2020, and the growth rate increased year by year, with a substantial increase of approximately 70 articles in 2017. Additionally, the number of articles from China and Germany exhibited remarkable growth compared with those from other countries (Figure 2A). Figure 2B shows the network visualization map for a total of 64 countries/regions collaborations, where the size of the circle represents the number of publications and the thickness of the connecting lines indicates the extent of collaboration between the countries/regions. The top collaborative countries/regions in the field of POD can be observed visually. The US was located at the core of the network and cooperated frequently with China, Germany, Canada, England, Japan, and other countries.

### Analysis of authors and institutions

The top 20 authors with the highest number of publications on POD from 2000 to 2020 are listed in Table 2. Among them, Inouye, Sharon K. was listed on top with the largest number of studies (*n* = 66) and the highest H-index (34) and proved to be the most influential professor in the field of POD. Marcantonio Edward was second, with 63 articles, followed by Jones Richard (*n* = 36), Fong Tamara (*n* = 23), and Xie, Zhongcong (*n* = 22). Interestingly, the top five ranked authors aforementioned and eight authors out of the top 20 shared the same affiliation, Harvard University; thus, it was likely the most influential institution in terms of research on POD.

**TABLE 2:** The top 20 authors with the highest number of publications on postoperative delirium (POD).

Name	Affiliations	Number of papers	H- index
Inouye, Sharon K.	Harvard University	66	34
Marcantonio, Edward R.	Harvard University	63	29
Jones, Richard N.	Harvard University	36	21
Fong, Tamara G.	Harvard University	23	16
Xie, Zhongcong	Harvard University	22	12
Neufeld, Karin J.	Johns Hopkins University	20	14
Leung, Jacqueline M.	University of California San Francisco	19	15
Avidan, Michael S.	Washington University	18	11
Ely, E. Wesley	Vanderbilt University	17	17
Schmitt, Eva M.	Harvard University	17	13
Brown, Charles H.	Johns Hopkins University	17	12
Sieber, Frederick E.	Johns Hopkins University	16	10
Alsop, David C.	Harvard University	15	11
Travison, Thomas G.	Harvard University	15	10
Spies, Claudia D.	Charite Medical University of Berlin	15	6
MacLullich, Alasdair M. J.	University of Edinburgh	14	9
Wang, Dong-Xin	Peking University	14	9
de Rooij, Sophia E.	University of Groningen	14	9
Pandharipande, Pratik P.	Vanderbilt University	13	10
Bellelli, Giuseppe	University of Milano-Bicocca	13	10

Harvard University appeared to be the most influential institution in the field of POD, which was further proven by its having the largest number of studies (*n* = 171), highest H-index (*n* = 46), and highest degree centrality (*n* = 191) among the top 20 most productive institutions (Table 3). Second, the University of California System, whose number of studies (*n* = 77) was less than half that of Harvard University, followed by Johns Hopkins University (*n* = 76). Overall, most institutions were based in the US (*n* = 12), while the rest were based in Netherlands (*n* = 2), China (*n* = 2), Canada (*n* = 2), England (*n* = 1), and Germany (*n* = 1).

**TABLE 3:** The top 20 institutes with the highest number of publications in postoperative delirium (POD).

Rank	Affiliations	Number of papers	Total citations	Mean citations/Paper	H-index	Degree centrality
1	Harvard Univ	171	6,042	35.33	46	191
2	UNIV of California System	77	2,341	30.40	27	50
3	Johns Hopkins Univ	76	2,223	29.25	32	91
4	Vanderbilt Univ	54	998	18.48	25	44
5	Duke Univ	54	1,167	21.61	19	80
6	Charite Univ Med Berlin	49	618	12.61	17	13
7	Univ Toronto	42	1,166	27.76	20	14
8	Yale Univ	39	988	25.33	20	32
9	Brown Univ	37	868	23.46	18	52
10	Univ Penn	34	3,229	94.97	16	63
11	Washington Univ	30	1,460	48.67	12	33
12	Univ Med Ctr Utrecht	29	611	21.07	18	8
13	Univ Amsterdam	28	1,278	45.64	18	8
14	Peking Univ	25	1,092	43.68	10	7
15	Purdue Univ	24	670	27.92	19	21
16	Univ Edinburgh	23	223	9.70	14	10
17	Capital Med Univ	23	677	29.43	5	10
18	Univ Manitoba	22	1,134	51.55	13	18
19	Mayo Clin	21	574	27.33	13	38
20	Univ Groningen	20	344	17.20	11	5

**FIGURE 3:**
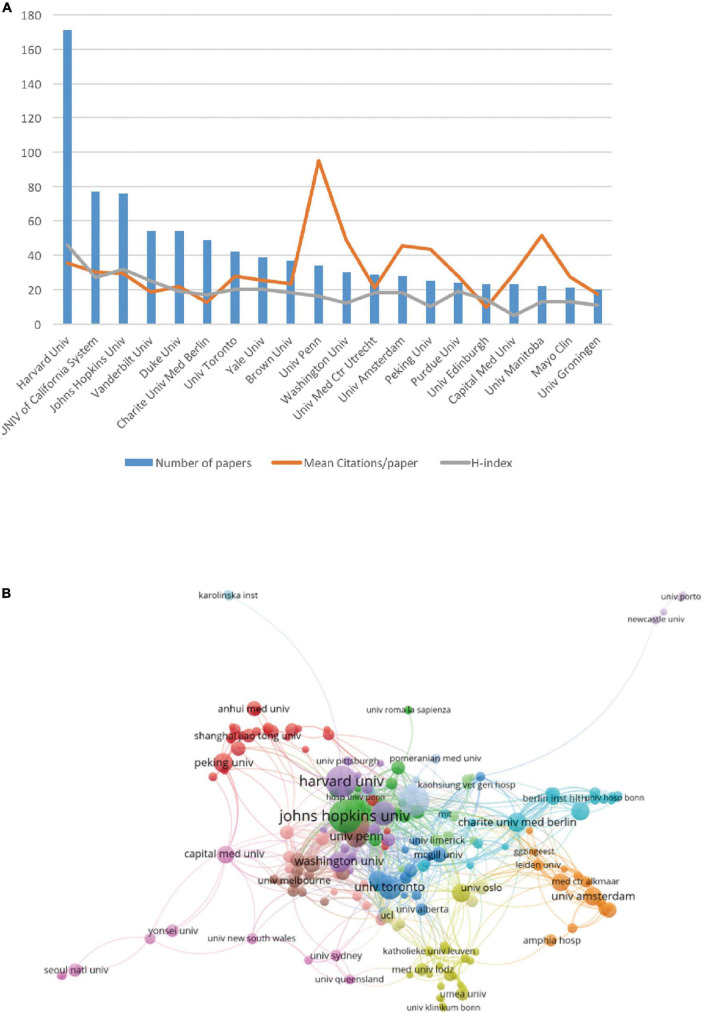
The articles on postoperative delirium (POD) published by different research institutions. **(A)** The top 20 most productive institutes. The blue bar graph represents the number of articles published by each institution, the red line represents the average number of citations per article, and the green line represents the H-index of each institution. **(B)** The network visualization map indicating collaborations between institutions.

Visually, the publication profile of the institutions is illustrated in Figure 3A. The number of studies from Harvard University clearly stands out among institutions making variation between other institutions looks like very small whose number of studies ranged from 20 to 77. Unlike the number of studies, the University of Pennsylvania ranked the highest in terms of the average number of citations (*n* = 94.97), followed by the University of Manitoba (*n* = 51.55), which ranked 10 and 18 out of 20, respectively. Next, we explored the cooperative relationships between institutions. As illustrated in Figure 3B, institutions worked closely with each other and were divided into several clusters according to their cooperation; however, no obvious boundaries were identifiable between these clusters, suggesting extensive collaboration worldwide. Moreover, Harvard University occupies the most important position in collaborative network.

### Analysis of journals

By analyzing the distribution of journals in the field of POD, we discovered that the *Journal of the American Geriatrics Society* published more articles (*n* = 80) than any other journal from 2000 to 2020, which were the most frequently cited, with 4,561 total citations and the highest average of 57.01; however, it only ranked sixth in terms of impact factor (IF) among the top 20 most productive journals (Table 4). Further, *Anesthesia and Analgesia* and the *British Journal of Anaesthesia* ranked second and third, with 52 and 43 publications, respectively, and their total and average citations were high among the top 20 journals. The top three journals belonged to Q1, a classification by Journal Citation Reports (JCR), representing their high status in their respective domains. Q1 journals had a higher rate of citations, such as the *International Journal of Geriatric Psychiatry* (average citation 28.09), *Anesthesiology* (average citation 37.94), the *American Journal of Geriatric Psychiatry* (average citation 41.93), and *Critical Care Medicine* (average citation 55.74).

**TABLE 4:** The top 20 journals with the highest number of publications on postoperative delirium (POD).

Rank	Journal	Number of papers	Total citations	Mean citations/ Paper	Impact factor (IF)	Journal Citation Reports (JCR)
1	Journal of the American Geriatrics Society	80	4,561	57.01	4.18	Q1
2	Anesthesia and Analgesia	52	1,807	34.75	4.305	Q1
3	British Journal of Anaesthesia	43	1,543	35.88	6.88	Q1
4	PLoS One	38	599	15.76	2.74	Q2
5	Journal of Cardiothoracic and Vascular Anesthesia	37	636	17.19	2.258	Q3
6	BMJ Open	36	433	12.03	2.496	Q2
7	International Journal of Geriatric Psychiatry	34	955	28.09	2.675	Q1
8	Anesthesiology	32	1,214	37.94	7.067	Q1
9	Current Opinion in Anesthesiology	32	337	10.53	2.276	Q3
10	American Journal of Geriatric Psychiatry	27	1,132	41.93	3.393	Q1
11	Journal of Clinical Anesthesia	25	194	7.76	6.039	Q1
12	Canadian Journal of Anesthesia-Journal Canadien D Anesthesie	24	373	15.54	3.779	Q2
13	Psychosomatics	23	727	31.61	2	Q2
14	Clinical Interventions in Aging	23	563	24.48	3.023	Q2
15	BMC Anesthesiology	23	159	6.91	1.695	Q4
16	General Hospital Psychiatry	22	769	34.95	2.86	Q2
17	Aging Clinical and Experimental Research	22	373	16.95	2.697	Q3
18	Medicine	22	64	2.91	1.552	Q3
19	Critical Care Medicine	19	1,059	55.74	7.414	Q1
20	Minerva Anestesiologica	19	387	20.37	2.498	Q3
21	Trials	19	143	7.53	1.883	Q3

**FIGURE 4:**
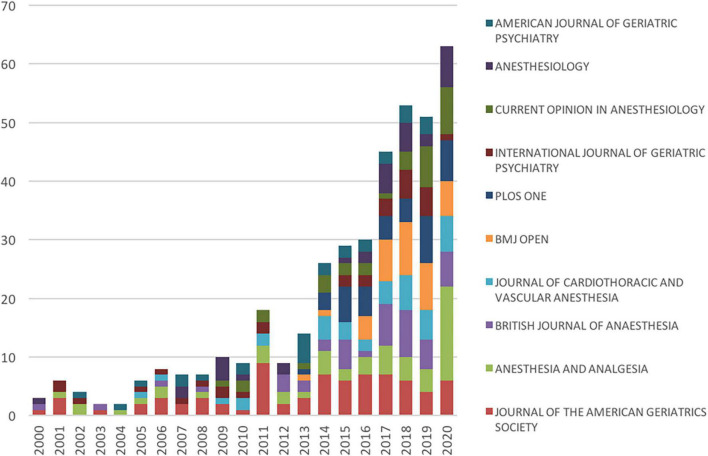
The number of articles on postoperative delirium (POD) published in the top 10 journals each year.

The number of studies published in the top 10 journals exhibited an overall growing trend, particularly in 2011, 2014, and 2017 (Figure 4). Notably, there was a visible increase in the number of papers published in *Anesthesia and Analgesia* in 2020.

### Analysis of funding agency and subject areas

Studies were supported by various funding agencies, which significantly motivated the development of POD research. The top 10 funding bodies are listed in Table 5, with the first three being the US Department of Health Human Services, National Institutes of Health (NIH), and NIH National Institute on Aging. The top three funding agencies funded 73.7% of the publications, and the number of funded papers exceeded 200. Among the remaining seven funding agencies, the National Natural Science Foundation of China listed the fourth with 67 founded articles. Overall, among these agencies, six were based in the US, two in Japan, one in China, and one in Europe.

**TABLE 5:** The top 10 funding agencies with the highest number of publications on postoperative delirium (POD).

Rank	Funding agency	Number of papers
1	United States Department of Health Human Services	333
2	National Institutes of Health NIH USA	327
3	NIH National Institute on Aging NIA	237
4	National Natural Science Foundation of China NSFC	67
5	NIH National Heart Lung Blood Institute NHLBI	53
6	NIH National Center for Advancing Translational Sciences NCATS	49
7	European Commission	42
8	Ministry of Education Culture Sports Science and Technology Japan MEXT	38
9	NIH National Institute of General Medical Sciences NIGMS	38
10	Japan Society for the Promotion of Science	32

**FIGURE 5:**
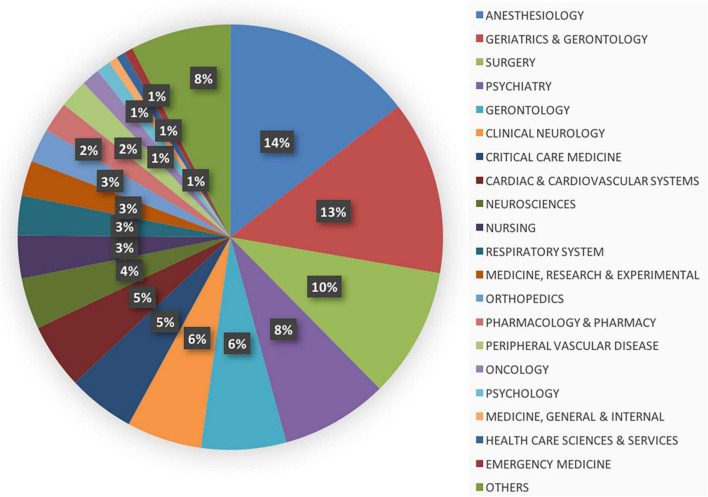
The distribution of top 20 subjects associated with the field of postoperative delirium (POD).

As aforementioned, POD is a complication associated with surgeries and anesthesia with a high incidence in the elderly, representing altered mental status; hence, the subject areas in the POD research field include *anesthesiology* (*n* = 471, 14%), *geriatrics and gerontology* (*n* = 431, 13%), *surgery* (*n* = 322, 10%), and *psychiatry* (*n* = 265, 8%), contributing to nearly half (45%) of the total articles (Figure 5). POD is highly prevalent after cardiac surgery and requires multidisciplinary management; therefore, other relevant subjects include *clinical neurology, critical care medicine, cardiac and cardiovascular systems, neuroscience, and nursing.*

### Analysis of the most frequently cited articles

**FIGURE 6:**
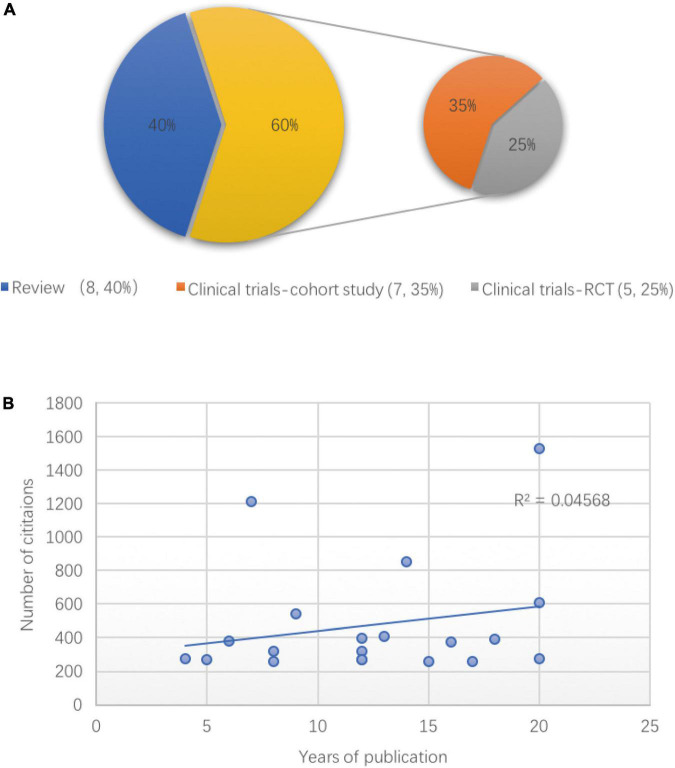
The classification and correlation analysis of the top 20 cited articles on postoperative delirium (POD). **(A)** The article types of the top 20 cited articles on POD, randomized controlled trial (RCT). **(B)** Linear correlation between the years of publication and numbers of citations among the top 20 POD articles.

The top 20 cited articles on POD are listed in Table 6, with citation counts ranging from 256 to 1,531. The classification of these publications is shown in Figure 6A. Of these, 12 articles were original research, all of which were clinical trials (*n* = 12, 60%), primarily cohort studies (*n* = 7, 35%), and randomized controlled trials (*n* = 5, 25%), and the remaining eight were reviews (systematic or literature reviews were defined as reviews) (*n* = 8, 40%), and the most cited article was written by *Ely, EW et al*., published by *JAMA*-*Journal of the American Medical Association* in 2001, and titled “Delirium in mechanically ventilated patients - Validity and reliability of the Confusion Assessment Method for the intensive care unit (CAM-ICU).” This prospective cohort study tested the validity and reliability of the CAM-ICU, which turned out to be “rapid, valid, and reliable for diagnosing delirium in the ICU setting (Ely et al., 2001b).” Another article published by the same author was ranked fourth (cited 606 times) out of the top 20. The second and third -most cited papers (cited 1,215 and 851 times) were published by the same author, Inouye et al. (2014b), both were reviews, entitled “Delirium in elderly people” and “Geriatric syndromes: Clinical, research, and policy implications of a core geriatric concept (Inouye et al., 2007).” Additionally, another article by *Inouye, Sharon K.* was included in the top 20. From the perspective of publication years, there were more articles published in the preceding decade, from 2000 to 2010 (*n* = 13). We then analyzed the association between the number of citations and the year of publication. However, the number of citations did not correlate with the year of publication (*R*^2^ = 0.0457, *p* = 0.366; Figure 6B). Additionally, not all articles were published in journals with high IFs, and most were from the US.

**TABLE 6:** The top 20 highest-cited articles on postoperative delirium (POD).

Rank	Title	Corresponding author	Affiliation	Source title	Year of publication	Cited by
1	Delirium in mechanically ventilated patients - validity and reliability of the confusion assessment method for the intensive care unit (CAM-ICU)	Ely, EW	Vanderbilt Univ	Jama-Journal of the American Medical Association	2001	1531
2	Delirium in elderly people	Inouye, Sharon K.	Univ Harvard Hebrew SeniorLife	Lancet	2014	1215
3	Geriatric syndromes: Clinical, research, and policy implications of a core geriatric concept	Inouye, Sharon K.;	Univ Harvard Hebrew SeniorLife	Journal of the American Geriatrics Society	2007	851
4	The impact of delirium in the intensive care unit on hospital length of stay	Ely, EW;	Vanderbilt Univ	Intensive Care Medicine	2001	606
5	Cognitive trajectories after postoperative delirium	Saczynski, Jane S.	Univ Massachusetts	New England Journal of Medicine	2012	542
6	The confusion assessment method: A systematic review of current usage	Inouye, Sharon K.	Univ Harvard Hebrew SeniorLife	Journal of the American Geriatrics Society	2008	406
7	Delirium in elderly adults: diagnosis, prevention and treatment	Fong, Tamara G.	Univ Harvard Hebrew SeniorLife	Nature Reviews Neurology	2009	396
8	Relationship between pain and opioid analgesics on the development of delirium following hip fracture	Morrison, RS	Mt Sinai Sch Med	Journals of Gerontology Series A-Biological Sciences and Medical Sciences	2003	394
9	A multicenter trial of remote ischemic preconditioning for heart surgery	Meybohm, P.	Univ Hosp Frankfurt	New England Journal of Medicine	2015	379
10	Haloperidol prophylaxis for elderly hip-surgery patients at risk for delirium: A randomized placebo-controlled study	Kalisvaart, KJ	Med Ctr Alkmaar	Journal of the American Geriatrics Society	2005	375
11	BIS-guided Anesthesia decreases postoperative delirium and cognitive decline	Chan, Matthew T. V.;	Chinese Univ Hong Kong	Journal of Neurosurgical Anesthesiology	2013	322
12	Postoperative delirium in the elderly risk factors and Outcomes	Robinson, Thomas N.	Univ of Colorado Denver Sch Med	Annals of Surgery	2009	320
13	European society of Anaesthesiology evidence-based and consensus-based guideline on postoperative delirium	Spies, Claudia D.	Charite Univ Med Berlin	European Journal of Anaesthesiology	2017	277
14	Use of medications with anticholinergic effect predicts clinical severity of delirium symptoms in older medical inpatients	Han, L	St Marys Hosp Ctr	Archives of Internal Medicine	2001	274
15	Derivation and validation of a preoperative prediction rule for delirium after cardiac surgery	Rudolph, James L.	VA Boston Healthcare Syst	Circulation	2009	272
16	Dexmedetomidine for prevention of delirium in elderly patients after non-cardiac surgery: a randomised, double-blind, placebo-controlled trial	Wang, Dong-Xin	Peking Univ, Hosp	Lancet	2016	270
17	The cognitive impact of anticholinergics: A clinical review	Boustani, Malaz;	Regenstrief Inst Inc.	Clinical Interventions in Aging	2009	268
18	The association between delirium and cognitive decline: A review of the empirical literature	Jackson, JC	Vanderbilt Univ	Neuropsychology Review	2004	260
19	Monitoring depth of anaesthesia in a randomized trial decreases the rate of postoperative delirium but not postoperative cognitive dysfunction	Spies, C. D.	Charite	British Journal of Anaesthesia	2013	257
20	Preoperative risk assessment for delirium after non-cardiac surgery: A systematic review	Dasgupta, Mondipa	St Joseph’s Htlh Care	Journal of the American Geriatrics Society	2006	256

### Analysis of keywords and hotspots

**FIGURE 7:**
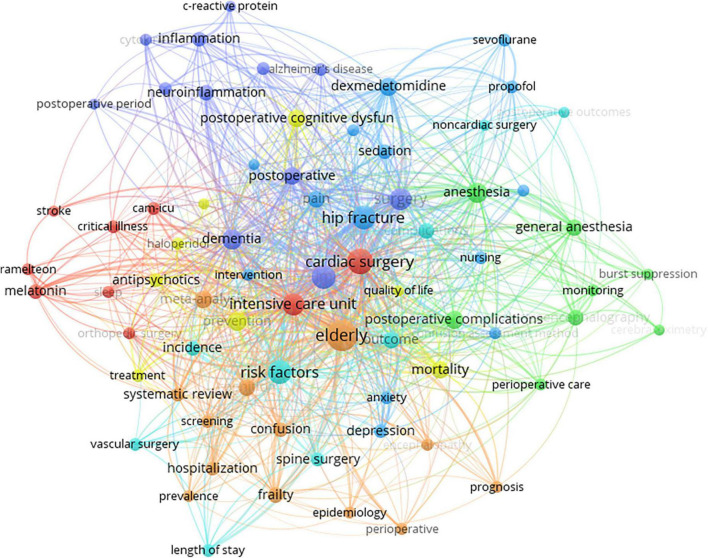
Map of hotspots and keyword co-occurrence analysis regarding postoperative delirium (POD).

Hotspots in the field of POD can be inferred by keyword co-occurrence analysis, which refers to the frequency of two keywords appearing together in the same article. Likewise, the size of the circles and the thickness of the line represent the frequency of occurrence and co-occurrence of keywords. As shown in Figure 7, “elderly” was the most frequently encountered keyword, indicating that studies of POD focus on the elderly, which is consistent with the high incidence of POD in the elderly. Second, the most frequent keywords co-occurring with “elderly” were “cardiac surgery,” “cognitive impairment,” “hip fracture,” and “intensive care unit.” Some sub-clusters were distinguished by different colors, which can be roughly divided into five groups: “anesthesia-related,” “drug-related,” “inflammation-related,” “prognosis-related,” and “cardiovascular surgery-related.” More specific research points are shown in Figure 7.

## Discussion

Delirium is an acute altered mental status, also be thought of as “acute brain failure,” with high incidence rates observed in the intensive care unit (Inouye et al., 2014b; Mattison, 2020). POD specifically refers to delirium that occurs during the postoperative period and is associated with poor clinical outcomes (Schenning and Deiner, 2015). Many studies have indicated that increasing age (>65 years) is a significant risk factor for POD (Steiner, 2011; Berger et al., 2018; Zhu et al., 2020), making POD a major concern in the context of global population aging, and an increasing number of articles about POD have been published. Here, we provide an overview of the developments in the field of POD over the past 20 years by applying bibliometric analysis, and 2,114 papers were identified and recruited from the Science Citation Index Expanded databases.

Overall, the number of papers on POD from various countries increased yearly, and frequent collaboration between countries suggested POD as a global issue. Among the countries, the US contributed the most due to having the most productive experts and institutions in the field of POD. And the core position of the US is mainly supported by several experts including Inouye, Sharon K., Marcantonio, Edward R., Jones, Richard N., Fong, Tamara G., and Xie, Zhongcong. Of these, Inouye appeared to be the most influential person with the highest number of publications and H-index. The first relevant study on delirium by Inouye et al. (1990) was published in 1990, after which he and his colleagues published many high-quality papers related to delirium or POD (Vasunilashorn et al., 2018) and developed a new delirium severity assessment, the Confusion Assessment Method-Severity (CAM-S) (Inouye et al., 2014a), which is widely used in clinical practice.

In terms of journals, the *Journal of the American Geriatrics Society* possessed both high quantity and quality of articles and is considered the most influential journal in the POD area indicating a good choice to publish researchers’ POD-related work or follow the frontier. While hotspot analysis results roughly reflect the different research branches at present, like elderly age, different anesthetics (sevoflurane, propofol, and dexmedetomidine) and different types of surgeries (cardiac surgery, non-cardiac surgery, hip fracture surgery, total knee arthroplasty, spine surgery, and vascular surgery). And inflammation, cytokines, and C-reactive protein, which are related to the biomarkers or mechanisms of POD, need to be explored in the future.

### Assessment tools

The top one and six highest-cited article were about the assessment tool for POD. CAM is the classical and the most widely used assessment tool for identification of POD, originally established in 1990 by Inouye et al. (1990). Also, there are other tools, such as the four “A”s Test (Shenkin et al., 2019), CAM-ICU13, EECHAM confusion scale (Van Rompaey et al., 2007), and intensive care delirium screening checklist (ICDSC) (Gusmao-Flores et al., 2012). Wilson et al. (2020) have summarized several common delirium screening tools which can provide reference for future studies.

### Epidemiology and identification of risk factors

Now we are aware that POD is a common global disease and is prevalent worldwide. But the incidence of POD varies greatly across different studies. In a 1-day point-prevalence study undertaken in 104 ICUs, the prevalence of POD was 32.3% (Salluh et al., 2010). And the overall incidence of POD was 22.9% in the Perioperative Neurocognitive Disorder and Biomarker Lifestyle (PNDABLE) study, which also showed that the higher the vascular risk score, the higher the incidence of POD (Wang et al., 2022). Likewise, in a meta-analysis, the top 20 cited article, the incidence of POD in included twenty-five studies ranged from 5.1 to 52.2%, especially those who have received hip fracture or aortic surgeries tend to have a higher rate (Dasgupta and Dumbrell, 2006). Consistent with findings in analysis of keywords and hotspots, older age (Robinson et al., 2009), cardiac surgery (Rudolph et al., 2009), hip fracture (Kalisvaart et al., 2005), etc., appear to be risk factors for POD, and the incidence of POD in intensive care unit was as high as 81.3% (Ely et al., 2001a), besides POD was associated with prolonged cognitive impairment (Jackson et al., 2004; Saczynski et al., 2012). In addition to the above−mentioned risk factors, there were other potential variables associated with the development of POD, including hypoalbuminemia, pre-existing dementia (Robinson et al., 2009), undertreated pain (Morrison et al., 2003), comorbidities (Yang et al., 2017), and etc.

### Prevention and therapy

Base on the identification of risk factors, targeted preventive measures can significantly reduce the incidence rate of POD. Several reviews (Fong et al., 2009; Janssen et al., 2019) have summarized some feasible interventions which can be broadly divided into preoperative (administration of melatonin, pre-operative multidisciplinary evaluation), intraoperative (infusion of dexmedetomidine, use of bispectral index (BIS)-guidance, varying tidal volumes during mechanical ventilation, choice of anesthetic regimen), postoperative (reducing postoperative pain, improving sleep, music therapy, orientation, oral and nutritional assistance, and early mobilization), or perioperative (multimodal intervention program), besides, there was no certain pharmacological therapy proved to be effective in improving POD except for Haloperidol. In short, identifying risk factors thereby adopt targeted measures for prevention was important for POD, and more effective medication should be explored in the future. We truly recommend the top 13 highest-cited article “European Society of Anaesthesiology evidence-based and consensus-based guideline on POD” (Aldecoa et al., 2017) published in 2017, which provides a comprehensive presentation of POD and practical recommendations in clinical practice.

### Basic research of postoperative delirium

From the analysis of the most frequently cited articles, it can be seen that basic research of POD is relatively sparse, partly due to the lack of suitable animal models. Some performed orthopedic surgery using aged Alzheimer’s disease transgenic mice to establish POD model (Wang et al., 2020), some performed abdominal surgery under isoflurane in old mice (Liufu et al., 2020). Basic research is undeniably important in obtaining a comprehensive understanding of the pathogenesis of POD, for example neuroinflammation (Velagapudi et al., 2019) and mitochondrial dysfunction (Lu et al., 2020) were reported to be involved in the development of POD, but the underlying mechanism of POD still remains to be elucidated.

Our study has some limitations. As aforementioned, POD is now included in PND; thus, our search strategy might not have been sufficiently comprehensive, and might have been insufficiently sensitive to find all relevant articles. Moreover, the earlier the articles published, the greater the number of citations. It is better to consider the time factor, although a correlation analysis between the years of publication of articles in the top 20 list and their number of citations suggested that the citation of articles was mainly dependent on the quality of the articles themselves, studies with larger sample sizes are needed to confirm this.

Finally, we conclude that research on POD is flourishing worldwide. The US has a relatively high academic impact because of its productive expertise and institutions in this field. Despite much research illustrating the diagnosis and management of POD in clinical practice, more basic research for the mechanism study of POD is needed.

## Data availability statement

The original contributions presented in this study are included in the article/supplementary material, further inquiries can be directed to the corresponding authors.

## Author contributions

XWL and YC helped perform the literature search and data acquisition. XWL and HM helped perform the manuscript preparation. XL and KY helped perform the data and statistical analysis. HM helped perform the secure funding. TL helped conceive and design the structure of this manuscript and revise the manuscript. All authors contributed to the article and approved the submitted version.

## References

[B1] Aldecoa C. Bettelli G. Bilotta F. Sanders R. D. Audisio R. Borozdina A. (2017). European society of anaesthesiology evidence-based and consensus-based guideline on postoperative delirium. *Eur. J. Anaesthesiol.* 34 192–214. 10.1097/EJA.0000000000000594 28187050

[B2] American Geriatrics Society Expert Panel on Postoperative Delirium in Older Adults (2015). Postoperative delirium in older adults: Best practice statement from the American Geriatrics Society. *J. Am. Coll. Surg.* 220 136–148.e1. 25535170 10.1016/j.jamcollsurg.2014.10.019

[B3] Bai J. Liang Y. Zhang P. Liang X. He J. Wang J. (2020). Association between postoperative delirium and mortality in elderly patients undergoing hip fractures surgery: A meta-analysis. *Osteoporos. Int.* 31 317–326. 10.1007/s00198-019-05172-7 31741024

[B4] Berger M. Schenning K. J. Brown C. H. IV Deiner S. G. Whittington R. A. Eckenhoff R. G. (2018). Best practices for postoperative brain health: Recommendations from the fifth international perioperative neurotoxicity working group. *Anesth. Analg.* 127 1406–1413. 10.1213/ANE.0000000000003841 30303868 PMC6309612

[B5] Chen S. Zhang Y. Dai W. Qi S. Tian W. Gu X. (2020). Publication trends and hot spots in postoperative cognitive dysfunction research: A 20-year bibliometric analysis. *J. Clin. Anesth.* 67:110012. 10.1016/j.jclinane.2020.110012 32942145

[B6] Dasgupta M. Dumbrell A. C. (2006). Preoperative risk assessment for delirium after noncardiac surgery: A systematic review. *J. Am. Geriatr. Soc.* 54 1578–1589. 10.1111/j.1532-5415.2006.00893.x 17038078

[B7] Ely E. W. Gautam S. Margolin R. Francis J. May L. Speroff T. (2001a). The impact of delirium in the intensive care unit on hospital length of stay. *Intens. Care Med.* 27 1892–1900. 10.1007/s00134-001-1132-2 11797025 PMC7095464

[B8] Ely E. W. Inouye S. K. Bernard G. R. Gordon S. Francis J. May L. (2001b). Delirium in mechanically ventilated patients: Validity and reliability of the confusion assessment method for the intensive care unit (CAM-ICU). *JAMA* 286 2703–2710. 10.1001/jama.286.21.2703 11730446

[B9] Evered L. Silbert B. Knopman D. S. Scott D. A. DeKosky S. T. Rasmussen L. S. (2018). Recommendations for the nomenclature of cognitive change associated with anaesthesia and surgery-2018. *Br. J. Anaesth.* 121 1005–1012. 10.1016/j.bja.2017.11.087 30336844 PMC7069032

[B10] Fong T. G. Tulebaev S. R. Inouye S. K. (2009). Delirium in elderly adults: Diagnosis, prevention and treatment. *Nat. Rev. Neurol.* 5 210–220. 10.1038/nrneurol.2009.24 19347026 PMC3065676

[B11] Gusmao-Flores D. Salluh J. I. Chalhub R. Quarantini L. C. (2012). The confusion assessment method for the intensive care unit (CAM-ICU) and intensive care delirium screening checklist (ICDSC) for the diagnosis of delirium: A systematic review and meta-analysis of clinical studies. *Crit. Care* 16:R115. 10.1186/cc11407 22759376 PMC3580690

[B12] Hong T. Feng X. Tong W. Xu W. (2019). Bibliometric analysis of research on the trends in autophagy. *PeerJ* 7:e7103. 10.7717/peerj.7103 31205825 PMC6556104

[B13] Hughes C. G. Boncyk C. S. Culley D. J. Fleisher L. A. Leung J. M. McDonagh D. L. (2020). American society for enhanced recovery and perioperative quality initiative joint consensus statement on postoperative delirium prevention. *Anesth. Analg.* 130 1572–1590. 10.1213/ANE.0000000000004641 32022748 PMC7379173

[B14] Inouye S. K. Kosar C. M. Tommet D. Schmitt E. M. Puelle M. R. Saczynski J. S. (2014a). The CAM-S: Development and validation of a new scoring system for delirium severity in 2 cohorts. *Ann. Intern. Med.* 160 526–533. 10.7326/M13-1927 24733193 PMC4038434

[B15] Inouye S. K. Studenski S. Tinetti M. E. Kuchel G. A. (2007). Geriatric syndromes: Clinical, research, and policy implications of a core geriatric concept. *J. Am. Geriatr. Soc.* 55 780–791. 10.1111/j.1532-5415.2007.01156.x 17493201 PMC2409147

[B16] Inouye S. K. van Dyck C. H. Alessi C. A. Balkin S. Siegal A. P. Horwitz R. I. (1990). Clarifying confusion: The confusion assessment method. A new method for detection of delirium. *Ann. Intern. Med.* 113 941–948. 10.7326/0003-4819-113-12-941 2240918

[B17] Inouye S. K. Westendorp R. G. Saczynski J. S. (2014b). Delirium in elderly people. *Lancet* 383 911–922. 10.1016/S0140-6736(13)60688-1 23992774 PMC4120864

[B18] Jackson J. C. Gordon S. M. Hart R. P. Hopkins R. O. Ely E. W. (2004). The association between delirium and cognitive decline: A review of the empirical literature. *Neuropsychol. Rev.* 14 87–98. 10.1023/B:NERV.0000028080.39602.17 15264710

[B19] Janssen T. L. Alberts A. R. Hooft L. Mattace-Raso F. Mosk C. A. van der Laan L. (2019). Prevention of postoperative delirium in elderly patients planned for elective surgery: Systematic review and meta-analysis. *Clin. Interv. Aging* 14 1095–1117. 10.2147/CIA.S201323 31354253 PMC6590846

[B20] Kalisvaart K. J. de Jonghe J. F. Bogaards M. J. Vreeswijk R. Egberts T. C. Burger B. J. (2005). Haloperidol prophylaxis for elderly hip-surgery patients at risk for delirium: A randomized placebo-controlled study. *J. Am. Geriatr. Soc.* 53 1658–1666. 10.1111/j.1532-5415.2005.53503.x 16181163

[B21] Liufu N. Liu L. Shen S. Jiang Z. Dong Y. Wang Y. (2020). Anesthesia and surgery induce age-dependent changes in behaviors and microbiota. *Aging* 12 1965–1986. 10.18632/aging.102736 31974315 PMC7053599

[B22] Lu Y. Chen L. Ye J. Chen C. Zhou Y. Li K. (2020). Surgery/Anesthesia disturbs mitochondrial fission/fusion dynamics in the brain of aged mice with postoperative delirium. *Aging* 12 844–865. 10.18632/aging.102659 31929114 PMC6977661

[B23] Mattison M. L. P. (2020). Delirium. *Ann Intern Med* 173 Itc49–Itc64. 10.7326/AITC202010060 33017552

[B24] Mi X. Wang X. Yang N. Han Y. Li Y. Liu T. (2021). Hundred most cited articles in perioperative neurocognitive disorder: A bibliometric analysis. *BMC Anesthesiol.* 21:186. 10.1186/s12871-021-01408-4 34215213 PMC8252303

[B25] Morrison R. S. Magaziner J. Gilbert M. Koval K. J. McLaughlin M. A. Orosz G. (2003). Relationship between pain and opioid analgesics on the development of delirium following hip fracture. *J. gerontol. Ser. A Biol. Sci. Med. Sci.* 58 76–81. 10.1093/gerona/58.1.M76 12560416

[B26] Raats J. W. van Eijsden W. A. Crolla R. M. Steyerberg E. W. van der Laan L. (2015). Risk factors and outcomes for postoperative delirium after major surgery in elderly patients. *PLoS One* 10:e0136071. 10.1371/journal.pone.0136071 26291459 PMC4546338

[B27] Robinson T. N. Raeburn C. D. Tran Z. V. Angles E. M. Brenner L. A. Moss M. (2009). Postoperative delirium in the elderly: Risk factors and outcomes. *Ann. Surg.* 249 173–178. 10.1097/SLA.0b013e31818e4776 19106695

[B28] Roldan-Valadez E. Salazar-Ruiz S. Y. Ibarra-Contreras R. Rios C. (2019). Current concepts on bibliometrics: A brief review about impact factor, Eigenfactor score, CiteScore, SCImago Journal Rank, Source-Normalised Impact per Paper, H-index, and alternative metrics. *Ir. J. Med. Sci.* 188 939–951. 10.1007/s11845-018-1936-5 30511320

[B29] Rudolph J. L. Jones R. N. Levkoff S. E. Rockett C. Inouye S. K. Sellke F. W. (2009). Derivation and validation of a preoperative prediction rule for delirium after cardiac surgery. *Circulation* 119 229–236. 10.1161/CIRCULATIONAHA.108.795260 19118253 PMC2735244

[B30] Saczynski J. S. Marcantonio E. R. Quach L. Fong T. G. Gross A. Inouye S. K. (2012). Cognitive trajectories after postoperative delirium. *N. Engl. J. Med.* 367 30–39. 10.1056/NEJMoa1112923 22762316 PMC3433229

[B31] Salluh J. I. Soares M. Teles J. M. Ceraso D. Raimondi N. Nava V. S. (2010). Delirium epidemiology in critical care (DECCA): An international study. *Crit. Care* 14:R210. 10.1186/cc9333 21092264 PMC3220001

[B32] Schenning K. J. Deiner S. G. (2015). Postoperative delirium in the geriatric patient. *Anesthesiol. Clin.* 33 505–516. 10.1016/j.anclin.2015.05.007 26315635 PMC4555984

[B33] Shenkin S. D. Fox C. Godfrey M. Siddiqi N. Goodacre S. Young J. (2019). Delirium detection in older acute medical inpatients: A multicentre prospective comparative diagnostic test accuracy study of the 4AT and the confusion assessment method. *BMC Med.* 17:138. 10.1186/s12916-019-1367-9 31337404 PMC6651960

[B34] Steiner L. A. (2011). Postoperative delirium. Part 1: Pathophysiology and risk factors. *Eur. J. Anaesthesiol.* 28 628–636. 10.1097/EJA.0b013e328349b7f5 21785356

[B35] Van Rompaey B. Bossaert L. Shortridge-Bagett L. Schuurmans M. Truijen S. (2007). A comparison of the confusion assessment method for the intensive care unit and the NEECHAM confusion scale in intensive care delirium assessment. *Crit. Care* 11(Suppl 2):419. 10.1186/cc5579

[B36] Vasunilashorn S. M. Fong T. G. Albuquerque A. Marcantonio E. R. Schmitt E. M. Tommet D. (2018). Delirium severity post-surgery and its relationship with long-term cognitive decline in a cohort of patients without dementia. *J. Alzheimers Dis.* 61 347–358. 10.3233/JAD-170288 29171992 PMC5714669

[B37] Velagapudi R. Subramaniyan S. Xiong C. Porkka F. Rodriguiz R. M. Wetsel W. C. (2019). Orthopedic surgery triggers attention deficits in a delirium-like mouse model. *Front. Immunol.* 10:2675. 10.3389/fimmu.2019.02675 31911786 PMC6918861

[B38] Wang J. Wang L. Tang X. Wang F. Liu S. Wu X. (2022). The relationship between cardiovascular disease risk score and postoperative delirium: The PNDABLE Study. *Front. Aging Neurosci.* 14:851372. 10.3389/fnagi.2022.851372 35800979 PMC9252852

[B39] Wang P. Velagapudi R. Kong C. Rodriguiz R. M. Wetsel W. C. Yang T. (2020). Neurovascular and immune mechanisms that regulate postoperative delirium superimposed on dementia. *Alzheimers Dement.* 16 734–749. 10.1002/alz.12064 32291962 PMC7317948

[B40] Wilson J. E. Mart M. F. Cunningham C. Shehabi Y. Girard T. D. MacLullich A. M. J. (2020). Delirium. *Nat. Rev. Dis. Primers* 6:90. 10.1038/s41572-020-00223-4 33184265 PMC9012267

[B41] Yang Y. Zhao X. Dong T. Yang Z. Zhang Q. Zhang Y. (2017). Risk factors for postoperative delirium following hip fracture repair in elderly patients: A systematic review and meta-analysis. *Aging Clin. Exp. Res.* 29 115–126. 10.1007/s40520-016-0541-6 26873816

[B42] Zhu C. Wang B. Yin J. Xue Q. Gao S. Xing L. (2020). Risk factors for postoperative delirium after spinal surgery: A systematic review and meta-analysis. *Aging Clin. Exp. Res.* 32 1417–1434. 10.1007/s40520-019-01319-y 31471892

